# The association of self-injurious behaviour and suicide attempts with recurrent idiopathic pain in adolescents: evidence from a population-based study

**DOI:** 10.1186/s13034-015-0069-0

**Published:** 2015-09-28

**Authors:** Julian Koenig, Rieke Oelkers-Ax, Peter Parzer, Johann Haffner, Romuald Brunner, Franz Resch, Michael Kaess

**Affiliations:** Clinic for Child and Adolescent Psychiatry, Centre of Psychosocial Medicine, University of Heidelberg, Heidelberg, Germany; Department of Psychology, The Ohio State University, Columbus, OH USA

**Keywords:** Pain, Adolescents, Self-injury, Suicide attempts

## Abstract

**Background:**

While several population-based studies report that pain is independently associated with higher rates of self-destructive behaviour (suicidal ideation, suicide attempts, and self-injurious behaviour) in adults, studies in adolescents are rare and limited to specific chronic pain conditions. The aim of this study was to investigate the link between self-reported idiopathic pain and the prevalence and frequency of self-injury (SI) and suicide attempts in adolescents.

**Methods:**

Data from a cross-sectional, school-based sample was derived to assess SI, suicide attempts, recurrent pain symptoms and various areas of emotional and behavioural problems via a self-report booklet including the Youth Self-Report (YSR). Adolescents were assigned to two groups (presence of pain vs. no pain) for analysis. Data from 5,504 students of 116 schools in a region of South Western Germany was available. A series of unadjusted and adjusted multinomial logistic regression models were performed to address the association of pain, SI, and suicide attempts.

**Results:**

929 (16.88%) respondents reported recurrent pain in one of three areas of pain symptoms assessed (general pain, headache, and abdominal pain). Adolescents who reported pain also reported greater psychopathological distress on all sub-scales of the YSR. The presence of pain was significantly associated with an increased risk ratio (RR) for SI (1–3 incidences in the past year: RR: 2.96; >3 incidences: RR: 6.04) and suicide attempts (one attempt: RR: 3.63; multiple attempts: RR: 5.4) in unadjusted analysis. Similarly, increased RR was observed when adjusting for sociodemographic variables. While controlling for psychopathology attenuated this association, it remained significant (RRs: 1.4–1.8). Sub-sequent sensitivity analysis revealed different RR by location and frequency of pain symptoms.

**Conclusions:**

Adolescents with recurrent idiopathic pain are more likely to report previous incidents of SI and suicide attempts. This association is likely mediated by the presence of psychopathological distress as consequence of recurrent idiopathic pain. However, the observed variance in dependent variables is only partially explained by emotional and behavioural problems. Clinicians should be aware of these associations and interview adolescents with recurrent symptoms of pain for the presence of self-harm, past suicide attempts and current suicidal thoughts. Future studies addressing the neurobiology underpinnings of an increased likelihood for self-injurious behaviour and suicide attempts in adolescents with recurrent idiopathic pain are necessary.

## Background

While suicide is one of the major causes of death in adolescence [1–4], recurrent idiopathic pain, in particular primary headache and abdominal pain, is of the leading somatic health issues among this age group [5]. Several studies in adults report that suffering from persistent pain is independently associated with higher rates of self-destructive behaviour, including suicidal ideation, suicide attempts, completed suicides, and self-injury (SI) (i.e., the intentional, self-directed act of injuring one’s own body tissue by cutting, burning etc. regardless of the suicidal intent). However, existing studies primarily focus on specific chronic pain conditions (e.g. migraine, arthritis, back pain) in clinical samples [6, 7]. Only a few population-based studies previously reported an increased risk for suicidal ideation and suicide attempts in adults with unspecific pain [7–10].

Studies on the link between the frequency of pain symptoms and suicidal ideation and/or suicide attempts in adolescents are rare [11–14]. To date, only two population-based studies from representative samples of adolescents on the association between suicidal behaviour and recurrent pain exist [13, 14]. One study [13] identified a higher frequency of suicidal ideation in adolescents (age 13–15) with migraine with aura, supporting previous evidence derived from a smaller community-based sample [12]. A recent longitudinal study on a representative sample of adolescents in the US reported that headaches and muscle aches are associated with a greater risk for suicide ideations but not suicide attempts after controlling for depressive symptoms [14]. While the study by Wang et al. [13] assessed suicidal ideation in adolescents with a specific chronic pain condition (i.e., migraine), the study by van Tilburg et al. [14] is the only population-based study that assessed self-reported, chronic pain conditions (i.e., headache, stomach ache or upset stomach, aches, pains, or soreness in muscles or joints) by occurrence (i.e., 5-point scale: never, just a few times, about once a week, almost every day, every day) and their association with suicidal behaviour. Specifically, the association of recurrent pain and SI in adolescents is largely unexplored.

While both suicidal and nonsuicidal SI (NSSI; i.e., deliberate SI without suicidal intent) often occur in the context of psychiatric conditions, the prevalence for NSSI in non-clinical samples, according to a recent meta-analysis, is 17.2% among adolescents, 13.4% among young adults, and 5.5% among adults [15]. Although definitions do differ concerning the SI intent, both NSSI and deliberate self-harm (DSH; i.e., SI including self-poisoning done with or without suicidal intent) have a comparable prevalence [16]. Psychopathological processes likely mediate the association of recurrent pain and SI. The leading hypothesis, emphasizes that the recurrent experience of pain leads to social withdraw (less engagement in everyday activities), that may further lead to depressive thoughts and subsequent SI as well as suicidal ideation. However, previous studies found that chronic pain, and specifically chronic headache, was associated with suicide ideation after controlling for depression [14], suggesting that depression alone is not capable to explain the increased likelihood of SI in those with recurrent pain.

Research has yet to establish the link between pain symptoms and SI. In particular, research on such association in adolescents seems important, given that adolescence is a critical period for the developmental psychopathology of SI associated disorders such as the borderline personality disorder. Drawing on data from a German cross-sectional, representative, school-based sample, we sought to replicate and extend previous findings in this age group [14], estimating the incidence of SI and suicide attempts in adolescents reporting recurrent pain. In line with existing evidence, we hypothesized, that the recurrent experience of pain is associated with an increased risk ratio of SI and suicide. While the previous longitudinal study only controlled for depression, here we aimed to control for a host of psychopathological symptoms, potentially mediating the link between pain, SI and suicide attempts.

## Methods

### Study population and design

Data for the present analysis was derived from the Heidelberg School Study [17, 18], which investigated a large, cross-sectional, school-based sample. Enrolment in the study took place in cooperation with the Heidelberg Public Health Service and the University of Education between October 2004 and January 2005. All schools in the Rhein-Neckar District were invited to participate. The Rhein-Neckar District is typical for geographically mixed populations in Germany and shows a representative distribution of types of schools and parental socioeconomic status [19]. This is further reflected by the sociodemographic characteristics of the present sample (Table 1) that, in agreement with statistics on the German population at this age [19], contained an equal number of female and male participants, of which the majority attended the Gymnasium followed by the Realschule and Hauptschule, and lived with both parents at the time of assessment. Of 121 schools contacted (n = 6,842), 116 agreed to participate. Five schools declined participation without providing further reasons. All ninth-grade students of the 116 participating schools (n = 6,534) were requested to take part in the study; 349 students were absent on the day of the assessment, 100 students did not return their questionnaires (N = 6,085). For the present analysis, participants with complete data on both dependent variables (suicide attempts and SI) were included (n = 5,504).

**Table 1:** Sample char acteristics

	Total	No pain		Recurrent pain	*P* value
n (%)	5,504 (100)	4,575 (83.12)		929 (16.88)	
Type of school, *n* (%)					<0.001
Hauptschule [reference]	1,573 (28.58)	1,269 (27.74)		304 (32.72)	
Realschule	1,804 (32.78)	1,459 (31.89)		345 (37.14)	
Gymnasium	1,978 (35.94)	1,727 (37.75)		251 (27.02)	
Foerderschule	149 (2.71)	120 (2.62)		29 (3.12)	
Gender, *n (%)*					<0.001
Male [reference]	2,680 (48.69)	2,427 (53.05)		253 (27.23)	
Female	2,609 (47.40)	1,970 (43.06)		639 (68.78)	
Item missing	215 (3.91)	178 (3.89)		37 (3.98)	
Housing situation: living with, *n* (%)					<0.001
Both parents [reference]	4,039 (73.38)	3,414 (74.62)		625 (67.28)	
Mother	674 (12.25)	546 (11.93)		128 (13.78)	
Father	99 (1.80)	81 (1.77)		18 (1.94)	
Mother and new partner	428 (7.78)	319 (6.97)		109 (11.73)	
Father and new partner	46 (0.84)	42 (0.92)		4 (0.43)	
Foster home	19 (0.35)	15 (0.33)		4 (0.43)	
With other person	49 (0.89)	36 (0.79)		13 (1.40)	
Item missing	150 (2.73)	122 (2.67)		28 (3.01)	
Year of birth, *n (%)*					0.062
1987	108 (1.96)	89 (1.95)		19 (2.05)	
1988	724 (13.15)	584 (12.77)		140 (15.07)	
1989 [reference]	2,776 (50.44)	2,286 (49.97)		490 (52.74)	
1990	1,882 (34.19)	1,604 (35.06)		278 (29.92)	
1991	13 (0.24)	11 (0.24)		2 (0.22)	
1992	1 (0.02)	1 (0.02)		0 (0.00)	
Self injury, *n* (%)					<0.001
Never [reference]	4,688 (85.17)	4,055 (88.63)		633 (68.14)	
1–3/year	598 (10.86)	408 (8.92)		190 (20.45)	
>3/year	218 (3.96)	112 (2.45)		106 (11.41)	
Suicide attempts, *n* (%)					<0.001
Never [reference]	5,074 (92.19)	4,323 (94.49)		751 (80.84)	
One	315 (5.72)	193 (4.22)		122 (13.13)	
Multiple	115 (2.09)	59 (1.29)		56 (6.03)	
General pain, *n (%)*					<0.001
Never [reference]	4,207 (76.44)	3,810 (83.28)		397 (42.73)	
Somewhat or sometimes true	949 (17.24)	765 (16.72)		184 (19.81)	
Very true or often true	258 (4.69)	0 (0.00)		258 (27.77)	
Item missing	90 (1.64)	0 (0.00)		90 (9.69)	
Headache, *n* (%)					<0.001
Never [reference]	2,852 (51.82)	2,727 (59.61)		125 (13.46)	
Somewhat or sometimes true	1,980 (35.97)	1,810 (39.56)		170 (18.30)	
Very true or often true	609 (11.06)	0 (0.00)		609 (65.55)	
Item missing	63 (1.14)	38 (0.83)		25 (2.69)	
Abdominal pain, *n* (%)					<0.001
Never [reference]	3,598 (65.37)	3,296 (72.04)		302 (32.51)	
Somewhat or sometimes true	1,534 (27.87)	1,254 (27.41)		280 (30.14)	
Very true or often true	337 (6.12)	0 (0.00)		337 (36.28)	
Item missing	35 (0.64)	25 (0.55)		10 (1.08)	
YSR scales, mean (SD)					
1. Withdrawn/depressed	3.00 (2.38)	2.79 (2.24)		4.07 (2.72)	<0.001
2. Somatic complaints	2.93 (2.69)	2.21 (1.99)		6.47 (2.87)	<0.001
3. Anxious/depressed	5.59 (4.54)	5.43 (3.69)		8.79 (4.96)	<0.001
4. Social problems	2.06 (1.95)	1.98 (1.87)		2.49 (2.22)	<0.001
5. Thought problems	1.45 (1.93)	1.27 (1.73)		2.32 (2.55)	<0.001
6. Attention problems	4.58 (2.73)	4.28 (2.61)		5.98 (2.85)	<0.001
7. Delinquent behavior	4.51 (3.16)	4.25 (2.98)		5.81 (3.65)	<0.001
8. Aggressive behavior	8.8 (5.23)	8.28 (4.96)		11.2 (5.78)	<0.001

### Measures

All measures were obtained using self-reports compiled in a self-report booklet, including diverse questions regarding (1) socio-demographic characteristics; (2) the frequency of suicidal attempts and SI, (3) the presence, frequency, and location of pain symptoms, as well as (4) psychopathology.

*Demographic variables* Demographic information including age and gender of participants was obtained, in addition to the school type and living situation at home. After 4 years of elementary school, the German school system branches into three types of secondary schools. The so-called “Hauptschule” (Secondary General School that takes 5 years after Primary School) prepares pupils for vocational training, whereas the “Realschule” (Intermediate Secondary School) concludes with a general certificate of secondary education after 6 years. Eight years of “Gymnasium” provide pupils with a general university entrance qualification. With respect to their living situation, participants were asked if they live with both parents, the mother or father only, the mother or father and his/her new partner, if they live in a foster home or with a person other than their mother/father.

*Dependent variables: suicidal attempts and self*-*injury* Two dependent variables were assessed by pertinent sections of the German version [20] of the schedule for affective disorders and schizophrenia for school-age children [21] that had been inserted into the self-report booklet; (1) the frequency of SI (e.g. cutting, burning, etc.) per year was assessed by the following response options: never, 1–3 times a year, or 4 times or more a year, and (2) total suicide attempts were assessed by the question “*Have you ever tried to take your own life*?” (no, once, 2–3 times, more than 3 times). The two later categories on suicide attempts (2–3 times and more than 3 times) were combined for subsequent analysis to explore the occurrence of a single versus multiple suicide attempts.

*Psychopathology* To control for a broad range of emotional and behavioural problems potentially associated with SI and suicidal attempts, the German version [22] of the Youth Self-Report (YSR) [23] a self-report version of the Child Behaviour Checklist (CBCL) [24] was administered. This self-report questionnaire consists of eight scales, including withdrawn/depressed (YSR-1), somatic complaints (YSR-2), anxious/depressed (YSR-3), social problems (YSR-4), thought problems (YSR-5), attention problems (YSR-6), delinquent behaviour (YSR-7), aggressive behaviour (YSR-8), and the summary scales of internalizing and externalizing problems as well as an YSR total score of emotional and behavioural problems. Psychopathology was included as covariate in adjusted analysis. We excluded the somatic complaints *(*YSR-2*)* subscale from all analyses, given that the independent pain variables were derived from it (see below). The depression-anxiety sub-scale of the YSR was further modified before analysis—two items addressing suicidal thoughts (item 91) and SI (item 18) were excluded to avoid inter-correlation with the dependent variables. Each of the excluded item scores was imputed using the mean of the remaining items. The excluded items highly showed significant correlations with our dependent variables (YSR-18 and SI: r = 0.663; YSR-18 and suicide attempts: r = 0.506; YSR-91 and suicide attempts: r = 0.440; YSR-91 and SI: r = 476).

*Independent variables: pain symptoms* Pain symptoms were assessed by the three pain-associated items out of the YSR somatic complains sub scale on physical problems without known medical cause scale (56a–h): 56a—aches or pains (not stomach or headache), 56b—headache and 56f—abdominal pain/stomach aches. Each item was scored 0 = not true, 1 = somewhat or sometimes true or 2 = very true or often true. To address the overall effect of recurrent pain independent of location, respondents, who reported pain very often in one of the three locations questioned, were assigned to a group (with pain). In later sensitivity analysis, pain items were treated independently in simultaneous analysis by location and severity of reported symptoms.

### Statistical analyses

Descriptive statistics were derived for the entire sample and groups based on the reporting of pain symptoms. Groups (no pain vs. with pain) were compared on all included variables using Chi Square tests and students t-tests for continuous variables where applicable. A series of multinomial logistic regression analysis was conducted to explore the association between predictor variables (pain) and the presence and frequency of suicide attempts and SI.

First, multinomial logistic regressions addressing the risk ratio (RR) for (1) SI, and (2) suicide attempts were calculated on associations by group assignment (pain vs. no pain). In sub-sequent sensitivity analysis, the frequency (not true, sometimes true, often true) of pain symptoms by location (general, headaches, abdominal pain) and the presence of SI (never, 1–3 times a year, or 3 or more incidents a year) and suicide attempts (never, one time, multiple times) was used. All variables were dummy-coded and the following categories were treated as reference: (1) no SI, and (2) no suicide attempts. Multinomial logistic regressions were calculated for each characteristic of the dependent variables. Similar, the frequency of pain symptoms was dummy coded, and the response category 0 (not true) was treated as the reference category when calculating relative RR and their according confidence intervals (95% CI). In sensitivity analysis, relative RR were estimated for pain symptoms occurring sometimes and very often (reference: none).

For all regression analysis, first, unadjusted models containing only the predictor variables were calculated, not controlling for any covariates. Second, regression models were adjusted for sociodemographic variables (gender, age, school type, and living situation at home). Third, models were further adjusted for indicators of psychopathology, including all sub-scales of the YSR, except for the somatic complaints sub scale. All regression models were further adjusted for the nested structure of children´s data (clustering within school) using the STATA vce(cluster) option [25]. With this option, the standard errors are adjusted for intragroup correlation on the school level, thus relaxing the usual requirement of the observations (children) to be independent within groups (schools). Hence, observations are assumed to be independent across groups (schools) but not necessarily within groups. All analyses were conducted using STATA 13 SE.

## Results

Adolescents reporting recurrent pain were of similar age compared to adolescents not reporting recurrent pain but differed on all other included sociodemographic variables (Table 1). Adolescents with pain reported greater emotional and behavioural problems on all subscales of the YSR. Recurrent pain was associated with increased RR for SI and suicide attempts in unadjusted and adjusted analysis (Table 2). Adjusting for psychopathological distress significantly attenuated the association of pain, SI and suicide attempts. However, all models still revealed more incidences of both SI and suicide attempts in individuals with recurrent pain.

**Table 2:** Risk ratios for suicide-attempts and self-injury by group (no pain vs. recurrent pain)

	M1: unadjusted	M2: adjusted	M3: fully-adjusted
Self-injury	RR (95% CI)	RR (95% CI)	RR (95% CI)
1–3 times per year	2.956 (2.423–3.606)***	2.367 (1.933–2.899)***	1.433 (1.154–1.780)***
>3 times per year	6.039 (4.440–8.215)***	4.570 (3.351–6.232)***	1.816 (1.264–2.608)***
Suicide attempts	RR (95% CI)	RR (95% CI)	RR (95% CI)
One suicide attempt	3.630 (2.840–4.640)***	2.833 (2.190–3.665)***	1.402 (1.075–1.829)*
Multiple suicide attempts	5.450 (3.809–7.799)***	4.077 (2.830–5.874)***	1.751 (1.178–2.603)**

Risk ratios expressed with respect to references categories for suicide attempts (no suicide attempt) and SI (no SI) in respondents with recurrent pain compared to those not reporting recurrent pain; Model 1: unadjusted, only adjusted for 116 clusters (schools); Model 2: adjusted for sociodemographic variables: gender, age, school type, and living situation at home; Model 3: further adjustment for all sub-scales of the YSR except for somatic complaints.

*** *p* < 0.001; ** *p* < 0.01; * *p* < 0.05.

The explained variance derived from pseudo R^2^ values from general models (Table 2) predicting suicide attempts, was 4.62% in unadjusted models (R^2^ = 0.0462), 8.14% in partially adjusted models (R^2^ = 0.0814), and 19.55% (R^2^ = 0.1955) in fully adjusted models. Similar the explained variance in SI was 4.20% in unadjusted models (R^2^ = 0.0420), 7.79% in partially adjusted models (R^2^ = 0.0779), and 19.61% (R^2^ = 0.1961) in fully adjusted models.

Sensitivity analysis showed that the association of pain and SI was independent of the location of pain symptoms in unadjusted models, with the exception of relatively weak association shown for headache symptoms. Furthermore, while most of these associations were robust when controlling for sociodemographic variables, they were no longer significant when adjusting for emotional and behavioural problems (Table 3). However, even in fully adjusted models, the association of very frequent general pain (other than headaches and abdominal pain) and 1–3 incidents of SI per year remained significant. Similar findings were observed for models explaining suicide attempts (Table 4). Here, the association of very frequent headaches, and abdominal pain (sometimes) with one suicide attempt, as well as the association of very frequent abdominal pain and multiple suicide attempts remained significant in fully adjusted models.

**Table 3:** Risk ratios for self-injury by severity of pain symptoms in different locations

	M1: Unadjusted	M2: Adjusted	M3: Fully-Adjusted
1–3 times per year	RR (95% CI)	RR (95% CI)	RR (95% CI)
General pain (sometimes)	1.661 (1.354–2.037)***	1.581 (1.278–1.956)***	1.121 (0.901–1.393)
General pain (very often)	2.577 (1.879–3.533)***	2.355 (1.720–3.224)***	1.565 (1.132–2.165)**
Headaches (sometimes)	1.252 (1.021–1.535)*	1.156 (0.941–1.420)	1.084 (0.874–1.344)
Headaches (very often)	1.869 (1.354–2.581)***	1.586 (1.142–2.203)**	1.286 (0.918–1.801)
Abdominal pain (sometimes)	1.679 (1.356–2.080)***	1.465 (1.179–1.820)***	1.184 (0.939–1.494)
Abdominal pain (very often)	2.675 (1.967–3.637)***	2.239 (1.631–3.074)***	1.413 (0.996–2.003)
>3 times per year	RR (95% CI)	RR (95% CI)	RR (95% CI)
General pain (sometimes)	1.967 (1.368–2.828)***	1.963 (1.361–2.831)***	1.107 (0.745–1.645)
General pain (very often)	3.150 (1.775–5.592)***	3.045 (1.719–5.395)***	1.382 (0.747–2.559)
Headaches (sometimes)	1.080 (0.789–1.480)	0.964 (0.700–1.326)	0.903 (0.643–1.268)
Headaches (very often)	2.450 (1.453–4.133)***	1.983 (1.196–3.287)**	1.366 (0.822–2.269)
Abdominal pain (sometimes)	1.986 (1.406–2.805)***	1.575 (1.103–2.247)*	1.078 (0.740–1.569)
Abdominal pain (very often)	5.097 (3.345–7.767)***	3.869 (2.516–5.948)***	1.631 (0.968–2.749)

Ratios are expressed with respect to references categories for SI (no SI) and pain symptoms (no pain in the respective location); models on pain variables include missings (no response); Model 1: unadjusted, only adjusted for 116 clusters (schools); Model 2: adjusted for sociodemographic variables: gender, age, school type, and living situation at home; Model 3: further adjustment for all sub-scales of the YSR except for somatic complaints.

*** *p* < 0.001; ** *p* < 0.01; * *p* < 0.05.

**Table 4:** Risk ratios for suicide attempts by severity of pain symptoms in different locations

	M1: unadjusted	M2: adjusted	M3: fully-adjusted
One suicide attempt	RR (95% CI)	RR (95% CI)	RR (95% CI)
General pain (sometimes)	1.767 (1.307–2.387)***	1.683 (1.241–2.283)***	1.135 (0.827–1.557)
General pain (very often)	2.658 (1.748–4.043)***	2.444 (1.618–2.692)***	1.423 (0.931–2.175)
Headaches (sometimes)	1.093 (0.768–1.556)	1.011 (0.717–1.426)	0.941 (0.662–1.339)
Headaches (very often)	2.552 (1.661–3.920)***	2.149 (1.407–3.283)***	1.568 (1.047–2.348)*
Abdominal pain (sometimes)	2.192 (1.636–2.939)***	1.910 (1.398–2.611)***	1.506 (1.083–2.093)*
Abdominal pain (very often)	2.314 (1.558–3.436)***	1.933 (1.275–2.930)***	1.023 (0.648–1.615)
Multiple suicide attempts	RR (95% CI)	RR (95% CI)	RR (95% CI)
General pain (sometimes)	2.028 (1.233–3.333)**	2.029 (1.217–3.384)**	1.267 (0.738–2.175)
General pain (very often)	2.840 (1.539–5.240)***	2.856 (1.561–5.225)***	1.360 (0.743–2.487)
Headaches (sometimes)	1.220 (0.751–1.982)	1.085 (0.655–1.797)	0.978 (0.589–1.623)
Headaches (very often)	1.805 (0.873–3.731)	1.472 (0.720–3.010)	1.040 (0.533–2.030)
Abdominal pain (sometimes)	1.716 (1.066–2.761)*	1.388 (0.841–2.293)	1.041 (0.620–1.749)
Abdominal pain (very often)	5.795 (3.424–9.806)***	4.468 (2.579–7.743)***	1.986 (1.126–3.505)*

Risk ratios from simultaneous estimates are expressed with respect to references categories for suicide attempts (no suicide attempt) and pain symptoms (no pain in the respective location) respectively; models on pain variables include missings (no response); Model 1: unadjusted, only adjusted for 116 clusters (schools); Model 2: adjusted for sociodemographic variables: gender, age, school type, and living situation at home; Model 3: further adjustment for all sub-scales of the YSR except for somatic complaints.

*** *p* < 0.001; ** *p* < 0.01; * *p* < 0.05.

## Discussion

Evidence supports a higher risk for suicidal ideation and suicide attempts in adults with recurrent pain in population-based studies [7–10, 26]. The present analysis attempted to investigate the relationship between self-reported pain symptoms, SI and suicide attempts in a large cross-sectional, school-based sample of 5,504 adolescents in Germany. In line with a previous study [14] we found recurrent pain to be associated with suicide attempts. The increased risk ratio for suicide attempts in those with recurrent pain is of a similar magnitude as reported by others (odd ratios: 1.2–2.1 [14]). Adding to the existing literature, this is the first study to show that the experience of recurrent pain is also associated with SI that in turn is considered a major risk factor for suicidal behaviour. Similar to the previous study, our findings did not vary as a function of age and gender in adjusted analysis [14] and were mainly robust when controlling for further sociodemographic factors (school type, living situation) as well as emotional and behavioral problems.

Within the sample studied, 16.88% (n = 929) reported recurrent idiopathic pain in at least one of the selected locations. In line with previous evidence that adolescents with recurrent symptoms of pain, such as headache or abdominal pain, are more likely to report greater psychopathological distress—in particular internalizing problems, such as depression or anxiety [27–30]—adolescents with recurrent idiopathic pain reported significant higher scores in all YSR syndrome scales compared to adolescents with no idiopathic pain.

While other found chronic headaches to be associated with suicide ideation after controlling for depression [14], our sensitivity analysis showed, that headaches were associated with the smallest RR for both SI and suicide attempts. Noteworthy, we found that abdominal pain is associated with the greatest RR for both, SI and suicide attempts. Given the unique association of abdominal pain and depression in children and adolescents, this finding warrants further investigation.

While the interpretation of pseudo R^2^ values carries limitations, they provide an estimate that about 4% of variance in SI and suicide attempts is explained by recurrent pain. Most interestingly, recurrent pain and sociodemographic characteristics show a similar amount of explained variance in these models.

Furthermore, our analysis showed that, despite the independent effect of pain on SI and suicidal, behavior this association is largely attenuated by emotional and behavioral problems that explained about 12% variance predicting SI and suicide attempts. While previous studies only controlled for depression, here we addressed a broad range of psychopathological problems. These results support the hypothesis that psychopathological distress at least may partly mediate the association of self-reported pain with SI and suicide.

While the present analysis is based on cross-sectional data and therefore prohibits the drawing of causal conclusions on the directional nature of the association of pain, SI and suicide attempts, at least two potential pathways are possible. First, and more favorable, it has previously been shown that psychopathology—specifically depression—is a consequence of chronic pain [31]. The recurrent experience of somatic pain is an “inescapable stressor” [32] leading to alterations of physiological regulatory systems and psychological distress. The continuous experience of such unavoidable stressor may have behavioral (i.e., social withdraw, avoidance) as well as both emotional and cognitive (i.e., catastrophizing) consequences, representing a vulnerable state for the development of psychopathological symptoms and disease. Ultimately, this may lead to an increased risk of SI and suicide attempts. As others have framed it, chronic pain may facilitate the development of a key risk factor for suicide: fearlessness about death [33]. In line with Joiner’s interpersonal theory of suicide [34], recurrent pain may increase distress and lead to a greater desire to die; thus, promoting SI and suicidal behavior.

However, it is also possible that psychopathology represents an antecedent for recurrent pain or at least the altered experience of everyday pain symptoms leading to SI and suicide. While there is no direct empirical evidence in support of one of these hypotheses yet, we reconfirmed the association of self-reported pain and suicide and for the first time were able to establish a link between self-reported pain and SI. Furthermore, our results lend support that while psychopathology sizably attenuates the association of pain, SI and suicide—it fails to explain all of the shared variance between these variable. Therefore, it is also possible that there is a shared diathesis linking pain and psychopathology that is associated with SI and suicidal behavior, in addition to other possible variables not included as covariates.

While we can only speculate on potential biological mechanism underlying these associations, we like to emphasize the notion of an involvement of the endogenous opiod system, given that both—pain and SI—are associated with altered function of this regulatory system. Nocks’ integrative model describes potential alterations of pain processing as a specific risk factor for SI—framed as the pain analgesia hypothesis [35]. Indeed, individuals engaging in SI show altered pain perception and analgesia during acts of SI [36], potentially due to the suggested alterations of the endogenous opioid system, that is, individuals engaging in NSSI have lower resting levels of β-endorphin and enkephalins. Since these neurotransmitters are released by injuries to body tissue, individuals engaging in SI may be more sensitive to opioid-mediated reward, that in turn may reduce negative affect (for a review see [37]). This divergent emotional and physical pain perception [38] is considered to play a crucial role in NSSI that is turn is a significant risk factor for suicidal behavior and their high co-occurrence suggests a common biology [39]. There is preliminary support for the hypothesis that chronic pain is initially associated with an up-regulation of endogenous opioid systems that becomes dysfunctional over time [40]. This endogenous opioid anti-nociceptive system dysfunction is associated with elevated acute and chronic pain sensitivity in chronic pain patients [41], who, compared to controls, show reductions in the capacity to activate the μ-opioid receptor system in acute pain [42].

Following such thought, the up-regulation of the opiod system to encounter endogenous demands of a body in pain, may present a biological pathway to blunted pain sensitivity. The behavioral drive to self-injure in order to find release from pain-related distressed is reinforced by the pain free experience of SI—that is likely in states of heightened endogenous opiod mediated analgesia. The individual may uphold such self-destructive behavior in the future based on such experience to face new episodes of pain and associated challenges to cope with recurrent pain. Such model—if proven by empirical evidence—would provide an explanation for the reported association of pain and SI. The transition from an initial up-regulation of the endogenous opioid system to its breakdown, the related changes in pain sensitivity, and the association of these alterations in physiological functioning with SI, bear promise to reveal some of the mechanism underlying the statistical associations we report. However, this is hypothetical and longitudinal studies are needed to allow conclusions on directionality and causality in the association between altered pain sensitivity, recurrent pain and SI. Assuming that alterations of the endogenous opioid system are best described as maladaptive long-term processes in those with chronic pain, adolescents with a recent onset of pain might be at an elevated risk given the initial changes in related physiology. However, there is no data in support of such idea, and the current data does not contain information on the pain history (e.g. age of onset) in the present sample. Future research is needed to test such hypothesis by exploring early alterations of pain sensitivity in those with recurrent pain, potentially leading to SI.

Finally, the present study has several noteworthy limitations. While we recruited our sample from all schools from a typical region in Germany, it was not random and may not be nationally representative. The school based study population excludes high-risk adolescents or adolescents with severe symptoms not attending school. As students who were absent on the day of the survey may be more likely to report more severe symptoms of pain (e.g. chronic headache) this is a potential source of bias. Furthermore, since the assessment was self-report based, students with language difficulties or learning disabilities may have been less likely to complete the questionnaires. Two further limitations are the definition of SI regardless of the suicidal intent (which is not according to current DSM-5-definitions), and the cross-sectional nature of the study not allowing for conclusions on directions of the observed relationships. While we assessed pain symptoms within the last 6 months based on the YSR, the assessment of dependent variables was based on lifetime incidents of SI and suicide attempts. However, we found that our dependent measures significantly correlated with the excluded items of the YSR addressing SI and suicide within the past 6 months. Finally, while we assessed the frequency of pain symptoms, we do not have any data on the quality of the painful experience, its duration and intensity. Future studies addressing these qualitative aspects asides measures of frequency may help to refine an understanding of individual differences in the experience of pain and its association with an increased risk to engage in SI and suicide. Furthermore, investigating the motivation for engaging in SI in those reporting recurrent pain symptoms may be useful in future studies.

## Conclusion

Adolescents with frequent idiopathic symptoms of pain are more likely to engage in SI and report more suicide attempts compared to their counterparts without likewise symptoms. Adolescents with recurrent idiopathic pain report more lifetime incidents of SI and suicide attempts, even when controlling for sociodemographic variables and psychopathology. Our results suggest that this association might be partially mediated by the severity and location of pain symptoms. Clinicians should be aware of the association between symptoms of pain and SI/suicide attempts and interview adolescents with recurrent symptoms of pain for the presence of self-harm or self-destructive-behaviour. Future research should include longitudinal studies to elucidate the directional relationship between symptoms of pain and different forms of self-destructive behaviour. Furthermore, studies are needed that will identify a common physiological mechanism underlying the association of recurrent pain, SI and suicide. Here we proposed that the endogenous opioid system might play a critical role that warrants further investigation.
